# A Study of High-Sensitivity Electro-Resistance Type Pre-Annealing ZnO-Doped CsPbBr_3_ Perovskite Acetone Sensors

**DOI:** 10.3390/s23042164

**Published:** 2023-02-14

**Authors:** Lung-Chien Chen, An-Ni Sung, Kun-Yi Lee

**Affiliations:** 1 Department of Electro-Optical Engineering, National Taipei University of Technology, Taipei 10608, Taiwan; 2 Department of Electrical Engineering, China University of Science and Technology, Taipei 11581, Taiwan

**Keywords:** perovskite, acetone gas sensor, CsPbBr_3_, pre-annealing ZnO

## Abstract

In this work, acetone gas sensors were fabricated using pre-annealing metal oxide zinc oxide (pa-ZnO)-doped perovskite cesium lead bromide (CsPbBr_3_). The ZnO nanopowder, before it was doped into CsPbBr_3_ solution, was first put into a furnace to anneal at different temperatures, and formed the pa-ZnO. The properties of pa-ZnO were different from ZnO. The optimized doping conditions were 2 mg of pa-ZnO nanopowder and pre-annealing at 300 °C. Under these conditions, the highest sensitivity (gas signal current-to-air background current ratio) of the ZnO-doped CsPbBr_3_ perovskite acetone sensor was 1726. In addition, for the limit test, 100 ppm was the limit of detection of the ZnO-doped CsPbBr_3_ perovskite acetone sensor and the sensitivity was 101.

## 1. Introduction

There are more than 3500 chemical components in the breath exhaled by the human body, most of which are volatile organic compounds (VOCs). Among the breath VOCs, acetone is a by-product of lipid metabolism that is closely related to blood glucose levels. Acetone in exhaled breath can thus monitor the metabolic state of the human body. Various techniques have been employed to measure the acetone of very low concentrations, such as gas chromatography–mass spectrometry, proton transfer reaction mass spectrometry, vacuum-free ion mobility spectrometry, laser absorption spectroscopy, and colorimetric sensors [1,2]. However, these techniques require bulky equipment and complex measurement procedures, making real-time monitoring impossible for widespread use throughout the human body. Therefore, it is of great significance to develop a convenient and low-cost method to accurately detect acetone at extremely low concentrations.

The boiling point and autoignition temperature of acetone are 56 °C and 465 °C, respectively. Metal oxide semiconductors are commonly used as sensor materials for sensing chemical gases, but they need to have good sensitivity in high-temperature environments, while perovskite can have a good gas sensing effect in room-temperature environments [3,4,5,6,7,8]. Halide perovskite materials have been widely used in light-emitting-diodes (LEDs) and photovoltaic devices due to their fascinating properties, including a high absorption coefficient, high photoluminescence quantum yield, and low non-radiative recombination rate [9,10,11,12,13,14], and as such, a lot of articles have published on LED and photovoltaic devices. In addition, owing to their excellent hydration–dehydration, electronic transition, adsorption–desorption, phase transition, and ion intercalaltion-decalationthe, the perovskite materials have a high sensitivity to the environment, such as temperature, humidity, VOC, etc., so perovskite materials are also suitable as environmental probes [15,16,17,18,19,20]. However, few articles have investigated their optoelectronic applications. Furthermore, acetone gas sensors made of zinc oxide (ZnO) had a good performance [8,21]. Therefore, in this study, complex materials of ZnO nanopowders and perovskites were prepared, and the ZnO nanopowders were annealed in a high-temperature furnace to increase their oxygen vacancies and conductivity, and then fabricated into a resistance-type acetone sensor with a high sensitivity when operated in room temperature.

## 2. Materials and Methods

**Figure 1:**
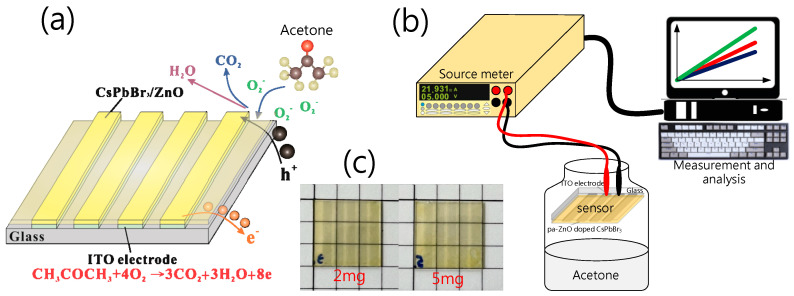
(**a**) Electro-resistance-type pa-ZnO-doped CsPbBr_3_ perovskite acetone sensor and sensing mechanism. (**b**) Structure of experiment system. (**c**) The photos of 2- and 5-mg pa-ZnO-doped CsPbBr_3_ perovskite films on ITO glass.

In the device preparation, the ZnO nanopowder with a size of 30 nm (Gredmann, 99.9%) was first put into a furnace to anneal at different temperatures, and formed the pa-ZnO. The second step was to prepare the pa-ZnO-doped CsPbBr_3_ solution. In total, 0.1835 g of PbBr_2_ powder (Alfa Aesar, 99.9999%), 0.1064 g of CsBr powder (Alfa Aesar, 99.9999%), and 1 mL of DMSO solvent (Alfa Aesar, 99.5%) were put into beaker and stirred with 500 ppm at a temperature of 70 °C for 30 min. Then, pa-ZnO nanopowders with various weights (1, 2, 5, 10 mg) were added into the beaker and stirred with 500 ppm at temperature of 70 °C for 1 day. Next, the third step was the formation of pa-ZnO-doped CsPbBr_3_ perovskite film for the acetone sensor. A total of 60 mL of pa-ZnO-doped CsPbBr_3_ solution was spin-coated on a glass substrate with an ITO (Ruilong, 7 ohm/cm^2^) pattern at 5000 ppm for 30 s. Additionally, the pa-ZnO-doped CsPbBr_3_ perovskite film was baked on a hot plate at 100 °C for 10 min to complete the electro-resistance-type pa-ZnO-doped CsPbBr_3_ perovskite acetone sensor, as shown in Figure 1a. 

In characteristic X-ray diffraction (XRD), a scanning electron microscope (SEM), photoluminescence (PL), UV/VIS spectrometers, and X-ray photoelectron spectroscopy (XPS) were used to measure and analyze the characteristics of pa-ZnO-doped CsPbBr_3_ perovskite films. Additionally, a Keithley 2400 source meter was employed to measure the I-V characteristics and to estimate the sensitivity of the electro-resistance-type pa-ZnO-doped CsPbBr_3_ perovskite acetone sensors. Figure 1b shows the structure of the experiment system for measurement. 

## 3. Results and Discussion

**Figure 2:**
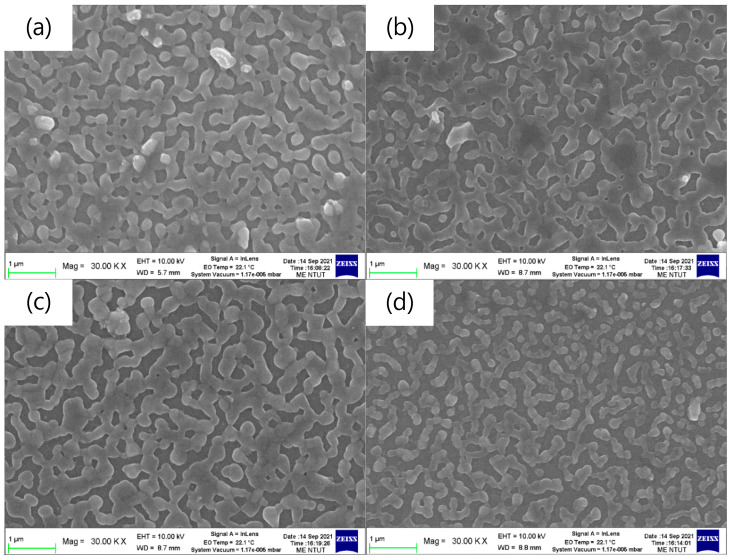
Top-view SEM images of the pa-ZnO-doped CsPbBr_3_ perovskite films on glass substrates with various amounts of pa-ZnO nanopowder: (**a**) 1 mg, (**b**) 2 mg, (**c**) 5 mg, and (**d**) 10 mg.

Figure 2 shows the top-view SEM images of the pa-ZnO-doped CsPbBr_3_ perovskite films on glass substrates. The coverages of all samples are around 83%, as estimated by Image J software. The aggregation domain sizes of the pa-ZnO-doped CsPbBr_3_ perovskite films, with 2 and 5 mg of pa-ZnO nanopowder doping, exhibited larger than that of the samples with 1 and 10 mg of pa-ZnO nanopowder doping, as show in Figure 2a–d. The pa-ZnO-doped CsPbBr_3_ perovskite films with 2 mg of the pa-ZnO nanopowder dopants had the largest aggregation domain size.

**Figure 3:**
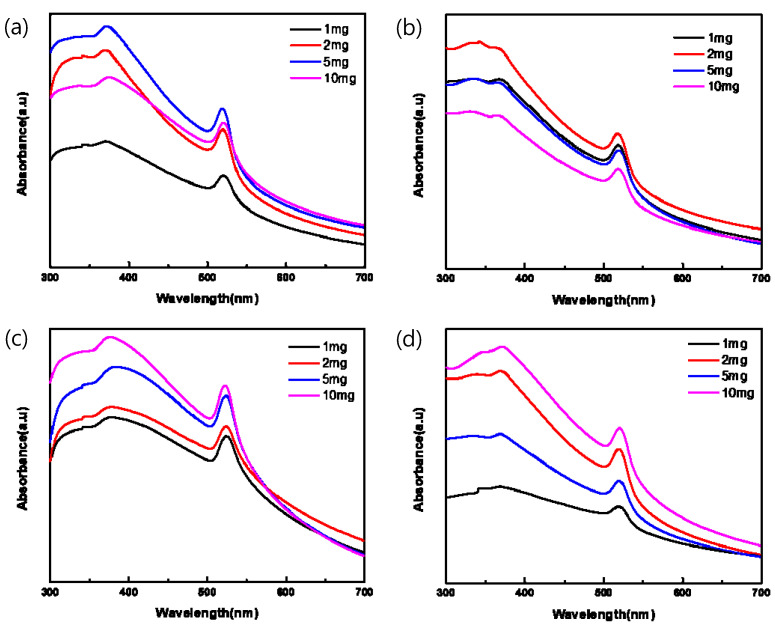
Absorption spectra of pa-ZnO-doped CsPbBr_3_ perovskite films with various amounts of pa-ZnO nanopowder dopants with treatment of annealing temperatures at (**a**) 200 °C, (**b**) 300 °C, (**c**) 400 °C, and (**d**) 500 °C.

Figure 3 plots the absorption spectra of the pa-ZnO-doped CsPbBr_3_ perovskite films, with various amounts of pa-ZnO nanopowder dopants with treatments of different annealing temperatures of 200–500 °C in UV-vis range. In total, two absorption peaks at 372 and 519 nm were observed. They are corresponding to the absorption of pa-ZnO and CsPbBr_3_. The peak position of the absorption did not shift with the doping amount of the pa-ZnO dopant and annealing temperature. This means that the nature of the materials of the pa-ZnO-doped CsPbBr_3_ perovskite films did not change. 

**Figure 4:**
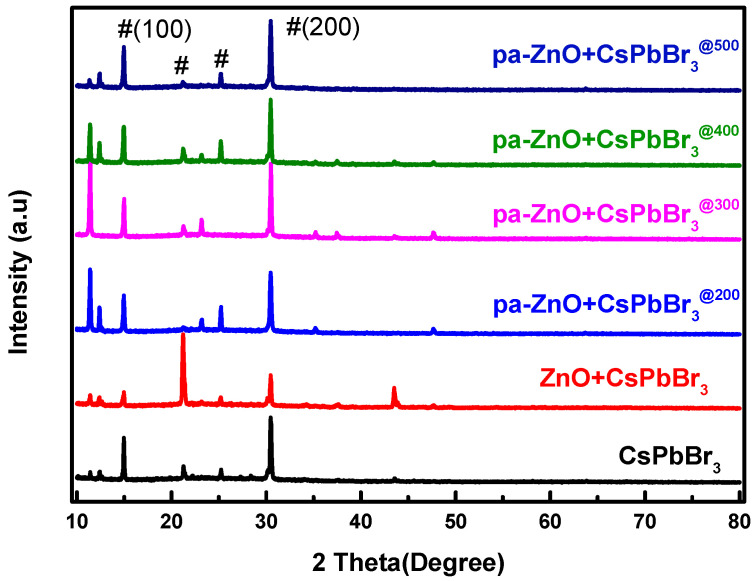
XRD pattern of pa-ZnO-doped CsPbBr_3_ perovskite films with various pre-annealing temperatures.

An XRD pattern was employed to understand the quality and composition variation of the pa-ZnO-doped CsPbBr_3_ perovskite films. As shown in Figure 4, there are four XRD characteristic peaks of perovskite CsPbBr_3_ that are observed. Their positions are at approximately 14.97°, 21.24°, 25.23°, and 30.47°, and are corresponding to the (100), (110), (111), and (200) phases of the cubic lattice structure, respectively [22,23,24]. A total of two weak diffraction peaks observed at the 2θ values of 35.24°and 37.45° have been indexed as (002) and (101) crystal planes of ZnO, respectively, for the sample of the pa-ZnO-doped CsPbBr_3_ perovskite film at an annealing temperature of 300 °C. This means that 300 °C is the optimized annealing condition. 

**Figure 5:**
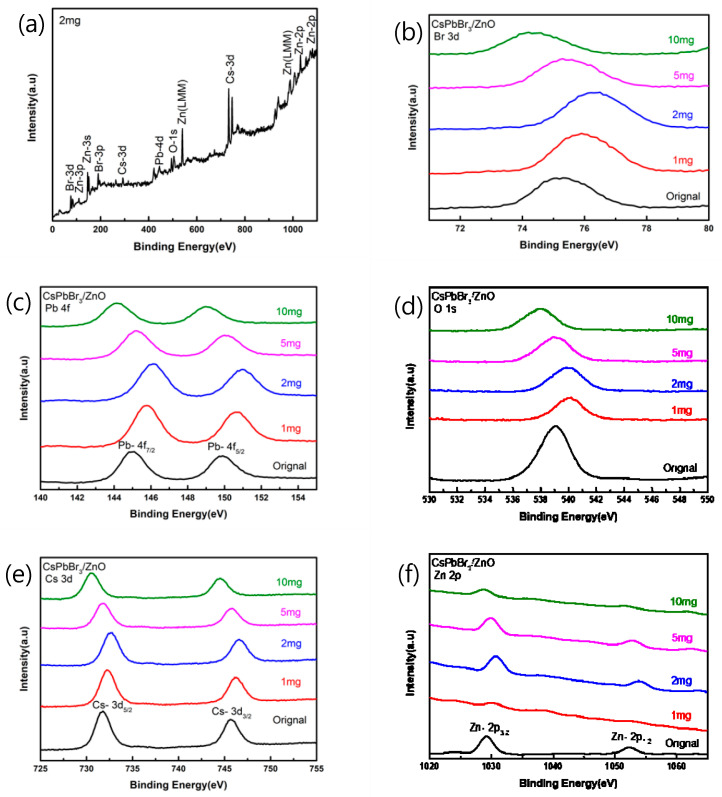
XPS spectra of pa-ZnO-doped CsPbBr_3_ films (**a**) added 2 mg of pa-ZnO nanopowder, and (**b**–**f**) in different binding energy range.

Figure 5a shows the XPS spectrum of CsPbBr_3_ added with 2 mg of pa-ZnO annealed at 300 °C. As shown in Figure 5b, the binding energy of Br-3d was increased from 75.21 eV of the original sample (undoped CsPbBr_3_ film) to 76.35 eV. As shown in Figure 5c, the binding energy of Pb-4f^5/2^ increased from 149.87 eV of the original sample to 150.97 eV, and the binding energy of Pb-4f^7/2^ increased from 144.97 eV of the original sample to 146.11 eV. As shown in Figure 5d, the binding energy of O-1s increased from 539.04 eV of the original sample to 540.11 eV. As shown in Figure 5e, the binding energy of Cs-3d^5/2^ increased from 731.68 eV of the original sample to 732.56 eV, and the binding energy of Cs-3d^3/2^ increased again from 745.65 eV of the original sample to 746.61 eV. As shown in Figure 5f, the binding energy of Zn-2p^3/2^ increased from 1029.22 eV of the original sample to 1030.78 eV. The binding energy of Zn-2p^1/2^ was increased from 1052.36 eV to 1054.07 eV to compare with the original sample. Therefore, it can be found that when 2 mg of pa-ZnO is added, for any different atoms, the binding energy is increased in comparison to the original sample, which is the highest in all samples. The phenomenon of this binding energy displacement may be that when the atoms are oxidized, the bond energy will be enhanced, and the oxidation will increase the binding energy of the inner layer electrons. The more electrons that are lost during oxidation, the greater this increase, and the peak position of the O-1s is related to the oxygen vacancies of the crystal lattice. It can be seen that, compared with the original sample of XPS spectra, the intensity after annealing has a downward trend, and it is known that the annealing heating causes oxygen vacancies [25,26].

**Figure 6:**
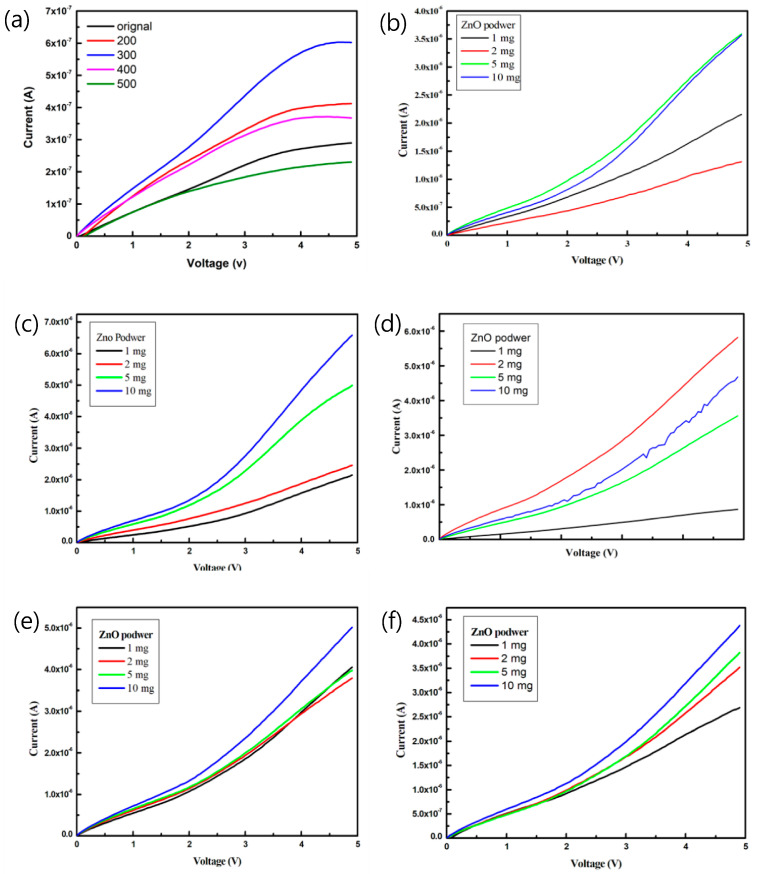
I–V characteristics of the pa-ZnO-doped CsPbBr_3_ perovskite acetone sensors with treatment of annealing at various temperatures and pa-ZnO doping amounts: (**a**) pa-ZnO sensor, (**b**) doping pa-ZnO without anneal, (**c**) doping pa-ZnO annealed at 200 °C, (**d**) doping pa-ZnO annealed at 300 °C, (**e**) doping pa-ZnO annealed at 400 °C, and (**f**) doping pa-ZnO annealed at 500 °C.

Figure 6 plots the current-voltage (I–V) characteristics of the pa-ZnO-doped CsPbBr_3_ perovskite acetone sensors with the treatment of annealing at various temperatures and pa-ZnO doping amounts. The sensitivity, S, can be used to estimate the performance of the gas sensor [5,27]:

(1) $$S=\frac{\left|I_{gas}-I_{air}\right|}{I_{gas}}$$

The sensitivity of the pa-ZnO-doped CsPbBr_3_ perovskite acetone sensors with the treatment of annealing at various temperatures and pa-ZnO doping amounts at 4.9 V, are summarized in Table 1. The sensitivity of the pa-ZnO-doped CsPbBr_3_ perovskite acetone sensors with the treatment of annealing at 300 °C and a doping amount of 2 mg shows the highest sensitivity, owing to the best conductivity, caused by a lot of oxygen vacancies. When the annealing temperature increases to 400 and 500 °C, the sensitivity decreases because of the description of oxygen vacancies, owing to the oxidation effect.

**Figure 7:**
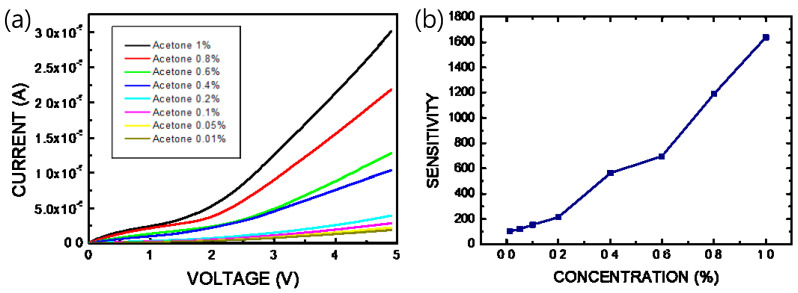
(**a**) I–V characteristics and (**b**) sensitivity of the pa-ZnO-doped CsPbBr_3_ perovskite acetone sensors with treatment of annealing at 300 °C and pa-ZnO doping amount of 2 mg under ambient of various concentration of acetone.

When the thin film of the pa-ZnO-doped CsPbBr_3_ perovskite is exposed to gas molecules to be measured, adsorbed oxygen on the pa-ZnO-doped CsPbBr_3_ perovskite film reacts with the acetone gas, thereby releasing electrons, as shown in Figure 1a. The electrons will fill the vacancies of the perovskite film and form electron and hole currents to improve the conductivity of the pa-ZnO-doped CsPbBr_3_ perovskite film. Therefore, we measured the current-voltage (I–V) characteristics to evaluate the performance of the pa-ZnO-doped CsPbBr_3_ perovskite acetone sensors. Figure 7 shows the I–V characteristics of the pa-ZnO-doped CsPbBr_3_ perovskite acetone sensors with the treatment of annealing at 300 °C and a pa-ZnO doping amount of 2 mg, under the ambient of various concentrations of acetone. Under the treatment of the champion condition: an annealing temperature of 300 °C and a pa-ZnO doping amount of 2 mg, the current of the pa-ZnO-doped CsPbBr_3_ perovskite acetone sensor decreases as the concentration of the acetone decreases. When the concentration of the acetone decreases to 0.01% (100 ppm), the I-V curve of the pa-ZnO-doped CsPbBr_3_ perovskite acetone sensor is a near-background I-V curve. Therefore, the 100 ppm is the limit of detection for the pa-ZnO-doped CsPbBr_3_ perovskite acetone sensor, and the sensitivity is 101. To compare the results of other works [4], the performance of the pa-ZnO-doped CsPbBr_3_ perovskite acetone sensor in this work have a competitive edge.

## 4. Conclusions

In this work, pa-ZnO-doped CsPbBr_3_ perovskite acetone sensors have been investigated. We have studied the morphology, absorption spectra, and XRD pattern of pa-ZnO-doped CsPbBr_3_ perovskite films for pre-annealing temperatures and doping amounts, respectively. The optimized pre-annealing temperature and doping amount are 300 °C and 2 mg of ZnO, respectively. According to the XPS spectra, the binding energy shifts with the amount of pa-ZnO nanopowder added. The effect this binding energy displacement may be that the atoms will be oxidized, the bond energy will be enhanced, and the oxidation will increase the binding energy of the inner layer electrons. Under the treatment of the champion condition, the highest sensitivity of the pa-ZnO-doped CsPbBr_3_ perovskite acetone sensor is 1726. In addition, 100 ppm is the limit of detection of the pa-ZnO-doped CsPbBr_3_ perovskite acetone sensor, and the sensitivity is 101. This work is not suitable for medical applications (1 ppm-level) [28,29,30,31]. However, the devices with an introduction of low-dimensional structure (quantum dots, nanorods, and nanosheets) may meet the requirement of biotechnology in the future.

## Figures and Tables

**Table 1:** Sensitivity of pa-ZnO-doped CsPbBr_3_ perovskite acetone sensors with treatment of annealing at various temperatures and pa-ZnO doping amounts at 4.9 V.

*pa-ZnO Doping*	w/o Annealing	200 °C	300 °C	400 °C	500 °C
1 mg	75	134	248	143	288
2 mg	351	755	1726	349	463
5 mg	512	951	1542	1250	430
10 mg	484	885	1158	1599	791

## Data Availability

The datasets used and/or analyzed during the current study are available from the corresponding author upon reasonable request.
